# *EGF*+61 A>G polymorphism does not predict response to first‐generation *EGFR* tyrosine kinase inhibitors in lung cancer patients

**DOI:** 10.1111/1759-7714.13628

**Published:** 2020-09-03

**Authors:** Leticia F. Leal, Ana C. Laus, Rodrigo Cavagna, Flavia E. de Paula, Marco A. de Oliveira, Dayana M. Ribeiro, Fernanda M. Hassan, José E. Miziara, Eduardo C. Albino da Silva, Vinicius D. da Silva, Pedro De Marchi, Rui M. Reis

**Affiliations:** ^1^ Molecular Oncology Research Center Barretos Cancer Hospital Barretos Brazil; ^2^ Barretos School of Health Sciences Dr. Paulo Prata – FACISB Barretos Brazil; ^3^ Molecular Diagnosis Center Barretos Cancer Hospital Barretos Brazil; ^4^ Department of Epidemiology and Biostatistics Barretos Cancer Hospital Barretos Brazil; ^5^ Department of Clinical Oncology Barretos Cancer Hospital Barretos Brazil; ^6^ Department of Surgery Barretos Cancer Hospital Barretos Brazil; ^7^ Department of Pathology Barretos Cancer Hospital Barretos Brazil; ^8^ Oncoclinicas Group Rio de Janeiro Brazil; ^9^ Life and Health Sciences Research Institute (ICVS) School of Medicine, University of Minho Braga Portugal; ^10^ ICVS/3B's – PT Government Associate Laboratory Braga Portugal

**Keywords:** *EGF*+61 A>G polymorphism, EGFR, first‐generation TKI SNP, lung cancer, TKI

## Abstract

Epidermal growth factor (EGF) and its receptor (EGFR) play a paramount role in lung carcinogenesis. The polymorphism in the *EGF* promoter region *EGF*+61A>G (rs4444903) has been associated with cancer susceptibility, but its role in lung cancer patients treated with tyrosine kinase inhibitors (TKIs) remains unknown. Here, we aimed to evaluate the predictive and prognostic role of *EGF*+61A>G SNP in lung cancer from Brazilian *EGFR*‐mutated TKI‐treated patients. Herein, patients carrying *EGFR*‐sensitizing mutations submitted to TKI treatment (gefitinib/erlotinib) were analyzed (*n* = 111) for *EGF*+61A>G genotype by TaqMan genotyping assay. TKI treatment was classified as partial response (PR), stable disease (SD), and disease progression (DP), according to RECIST1.1. Association analysis was assessed by chi‐square and Fisher's test (univariate) and multinomial model (multivariate) and survival analysis by Kaplan‐Meier method and log‐rank test. The *EGF*+61A>G genotype frequencies observed were: AA = 31.5% (*n* = 35), AG = 49.6% (*n* = 55) and GG = 18.9% (*n* = 21). The allelic frequencies were 56.3% for A, and 43.7% for G and the population was in Hardy‐Weinberg equilibrium (*P* = 0.94). *EGF*+61A>G codominant model (AA vs. AG vs. GG) was associated with a response to TKIs (*P* = 0.046), as well as a recessive model (AA vs. AG + GG; *P* = 0.023). The multinomial regression showed an association between the codominant model (AG) and recessive model (AG + GG) with SD compared with DP (*P* = 0.01;OR = 0.08; 95% CI = 0.01–0.60 and *P* = 0.02;OR = 0.12; 95% CI = 0.20–0.72, respectively). No association between genotypes and progression‐free or overall survival was observed. In conclusion, the *EGF*+61 polymorphism (AG and AG + GG) was independently associated with stable disease in lung cancer patients although it was not associated with the overall response rate to first‐generation TKIs or patient outcome.

## Graphical abstract

• *EGF*+61 SNP was assessed in TKI‐treated NSCLC patients harboring *EGFR*‐sensitizing mutations.

• *EGF*+61 SNP (AG + GG) was independently associated stable in *EGFR*‐mutated TKI‐treated NSCLC patients.

• *EGF*+61 SNP (AG + GG) cannot predict progression‐free and overall survival or overall response rate to first generation TKIs.

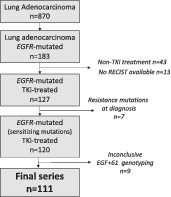

## Introduction

Epidermal growth factor (EGF) and its receptor (EGFR) play a key role in the pathogenesis of several tumors, including non‐small cell lung cancer (NSCLC).^1^, ^2^ EGFR is a critical therapeutic target, and the presence of *EGFR* mutations has previously successfully guided clinical management in NSCLC.^3^, ^4^ *EGFR* mutations located at the tyrosine kinase domain, sensitizes NSCLC to treatment with anti‐EGFR tyrosine kinase inhibitors (TKIs), such as erlotinib and gefitinib.^3^, ^4^, ^5^, ^6^ However, most TKI‐treated patients will experience disease progression due to resistance mechanisms, such as the presence of *EGFR* resistance mutations.^7^

A single nucleotide polymorphism (SNP) in the 5′ untranslated region of the *EGF* gene (*EGF*+61 A>G — rs4444903) has been associated with increased levels of EGF and consequently is a risk for cancer development of distinct tumors.^2^ In lung cancer, conflicting results have been reported in different populations.^8^, ^9^, ^10^, ^11^ Importantly, in advanced rectal cancer, EGF+61 A>G SNP predicted a complete pathologic response to cetuximab, an anti‐EGFR monoclonal antibody.^12^

Although cetuximab and TKIs are two different forms of therapies directed at *EGFR*, they both result in blocking signal transduction.^13^ Thus, we hypothesized that *EGF*+61 A>G SNP might be associated with a response to first‐generation TKIs in lung cancer. Thereafter, we investigated the association between *EGF*+61 A>G genotype and TKI response in a Brazilian series comprised of 111 TKI‐treated lung adenocarcinoma patients harboring *EGFR*‐sensitizing mutations.

## Methods

### Study population

This was a retrospective and observational study, which included patients with NSCLC, diagnosed between 2001 and 2017 at Barretos Cancer Hospital. The present study was approved by the Barretos Cancer Hospital IRB (Project No. 630/2012), and all methods were performed following the Helsinki declaration.

**Figure 1:**
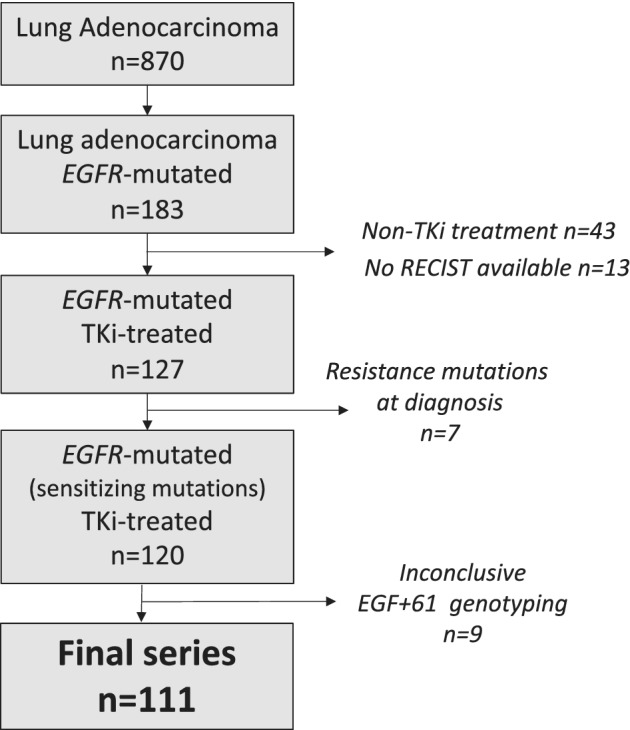
Workflow of sample procurement according to eligibility criteria.

From a series of lung adenocarcinoma cases (*n* = 870), 183 patients presented with *EGFR* mutations and 127 out of 183 patients were TKI‐treated (Fig 1). The response to TKI‐treatment was determined using RECIST 1.1, and the clinical response was categorized as partial response (PR) for overall response rate (ORR), stable disease (SD) for disease control rate (DCR), or disease progression (DP). NSCLC patients harboring *EGFR* resistance mutations (eg, T790M) are not currently eligible for erlotinib, and gefitinib treatment; thus, these patients (*n* = 7) were not evaluated (Fig 1). Nine patients were excluded due to inconclusive genotyping results **(**Fig 1 **)**. The demographic and clinicopathological data from eligible patients (*n* = 111) were obtained by reviewing the medical records (Table 1).

**Table 1:** Demographic and clinicopathological features of *EGFR*‐mutated TKI‐treated NSCLC patients enrolled in the study (*n* = 111)

Characteristics		*n* = 111	Frequencies
Sex	Male	47	42.3%
	Female	64	57.7%
Age	Median (SD)	61.75 (11.48)	
	≤63 years	58	52.3%
	>63 years	53	47.7%
Self‐reported skin color	White	96	86.5%
	Other	15	13.5%
Smoking status	Never	65	58.6%
	Current	17	15.3%
	Former	28	25.2%
	NA	1	0.9%
ECOG PS	0–1	78	70.3%
	2	20	18.0%
	3–4	8	7.2%
	NA	5	4.5%
Weight loss in the last six months prior to diagnosis	>10%	18	16.2%
	<10%	46	41.4%
	None	39	35.1%
	NA	8	7.3%
TNM stage	I/II/III	12	10.8%
	IV	99	89.2%
Histological subtype	Acinar	42	37.8%
	Mucinous	1	0.9%
	Lepidic	8	7.3%
	Papillary	10	9.0%
	Solid	10	9.0%
	Others	7	6.3%
	Not defined	33	29.7%
Central nervous system metastasis	Yes	32	28.8%
	No	79	71.2%
Current status	Alive	24	21.6%
	Dead due to cancer	80	72.1%
	Dead due to comorbidity	1	0.9%
	NA	6	5.4%
Response to TKI treatment	Partial response	78	70.3%
	Stable disease	18	16.2%
	Disease progression	15	13.5%
*EGFR* mutation according to exon location	Exon 19	69	62.2%
	Exon 20	3	2.7%
	Exon 21	25	22.5%
	Exon 18 and exon 21	2	1.8%
	Exon 19 and exon 20	9	8.1%
	Exon 20 and exon 21	3	2.7%

NA, data unavailable; SD, standard deviation.

### DNA isolation and EGFR mutational status

*EGFR* mutational status was assessed by examining formalin‐fixed paraffin‐embedded (FFPE) tumor tissue from NSCLC patients. DNA was isolated using QIAamp DNA Micro Kit (Qiagen) according to the manufacturer's instructions, and *EGFR* mutations were evaluated by Sanger sequencing as previously described.^14^ A subset (*n* = 444) of this series comprised of *EGFR*‐mutated patients has been previously reported.^14^

### *EGF*+61 A>G genotyping

*EGF*+61 A>G polymorphism was analyzed in the same DNA samples used to access *EGFR* mutation. Genotyping was performed using quantitative real‐time PCR, with a commercially available TaqMan genotyping assay (ThermoFisher, USA) (C_27031637_10 [*EGF*+61 A>G]) in the QuantStudio 6 Flex Real‐Time PCR System (ThermoFisher, USA) and under standard cycling, as previously reported.^8^

### Statistical analysis

Categorical variables are presented as percentages. Hardy‐Weinberg equilibrium was verified. *EGF*+61 A>G genotype was analyzed using three different models: codominant model – AA versus AG versus GG; recessive model – AA versus AG + GG; dominant model – AA+AG versus GG. χ^2^ test was applied to verify the association between TKI treatment response and *EGF*+61 A>G genotype and demographic and clinicopathological characteristics. The Kaplan‐Meier method and log‐rank test were employed for survival analysis, considering for progression‐free survival (PFS), disease progression as the event of interest and, for overall survival (OS), death as the event of interest. Patients who had not experienced disease progression were considered as censored for PFS and alive patients and those lost to follow up were considered as censored for OS.

Multinomial regression adjusted by age, TNM and PS‐ECOG were used to access the association between *EGF*+61 A>G genotype and TKI treatment response by computing the odds ratio (ORs) with 95% confidence interval (CI) considering DP as the reference category. All statistical analyses were performed using IBM SPSS Statistics, version 21.0 (IBM Corp, Armonk, NY, USA). A *P*‐value lower than 0.05 was considered statistically significant.

## Results

The demographic and clinicopathological characteristics of the 111 eligible patients (Fig 1) are detailed in Table 1. The median age was 61.75 years (SD ± 11.48), 47 were males and 64 females, most patients were never smokers (*n* = 65), and the majority were diagnosed at disease stage IV (*n* = 99) (Table 1). Notably, most patients harboring *EGFR*‐sensitizing mutations presented a partial response as the best TKI response (*n* = 78, 70.3%; Table 1), and none of the patients presented with a complete response (CR) (Table 1). The association between response to TKI treatment and demographic and clinicopathological characteristics from TKI‐treated NSCLC patients harboring *EGFR*‐sensitizing mutations (*n* = 111) was also assessed, and no association was revealed (Table S1).

*EGF*+61 A>G genotypic frequencies were 31.5% (*n* = 35) for AA, 49.6% (*n* = 55) for AG and 18.9% (*n* = 21) for GG. The allelic distribution was 56.3% for the A allele and 47.7% for the G allele (Table S2). The present series (*n* = 111) was in Hardy‐Weinberg equilibrium, according to *EGF*+61 A>G polymorphism (*P* = 0.941).

The patients were grouped into three categories according to response to treatment (PR vs. SD vs. DP) and three genotypic models to assess the association between *EGF*+61 A>G polymorphism and response to TKI treatment. There was an association between response to TKI treatment and *EGF*+61 A>G polymorphism for a codominant and recessive model (*P* = 0.046 and *P* = 0.023, respectively; Table 2). No association was observed between response to TKI treatment and the dominant model (*P* = 0.601, Table 2). In addition, no association was observed between response to TKI treatment and allele frequencies (*P* = 0.163) (Table 2).

**Table 2:** Association between *EGF*+61 genotype and alleles from TKI‐treated NSCLC patients harboring *EGFR*‐sensitizing mutations (*n* = 111) and TKI treatment response

	PR		SD		DP		
Response to TKI treatment	n	%	n	%	n	%	*P*‐value
Genotype (codominant model)							
AA	23	29.5%	10	55.6%	2	13.3%	**0.046**
AG	42	53.8%	4	22.2%	9	60.0%	
GG	13	16.7%	4	22.2%	4	26.7%	
Genotype (recessive model)							
AA	23	29.5%	10	55.6%	2	13.3%	**0.023**
AG + GG	55	70.5%	8	44.4%	13	86.7%	
Genotype (dominant model)							
AA + AG	65	83.3%	14	77.8%	11	73.3%	0.601
GG	13	16.7%	4	22.2%	4	26.7%	
Allele frequency							
A		56.4%		66.7%		43.3%	0.163
G		43.6%		33.3%		56.7%	

Bold *P*‐values indicate *P* ≤ 0.05 from univariate analysis (χ^2^ test).

DP, disease progression; PR, partial response; SD, stable disease.

Since *EGF*+61 A>G genotypes (codominant and recessive model) were associated with response to TKI treatment, we assessed whether any clinical parameter could be influenced by this result as a confounder variable and a multivariate analysis (multinomial analysis) was conducted adjusted for age, TNM, and PS‐ECOG. We observed that in the codominant model, AG genotype was independently associated with SD (*P* = 0.01; OR = 0.08; 95% CI: 0.01–0.60). Likewise, we observed that in the recessive model, AG + GG genotypes were independently associated with SD (*P* = 0.02; OR = 0.12; 95% CI: 0.20–0.72) (Table 3).

**Table 3:** Multivariate analysis for association between *EGF*+61 genotype and alleles from TKI‐treated NSCLC patients harboring *EGFR*‐sensitizing mutations (*n* = 111) and TKI treatment response

	Response to TkI treatment*		n	OR	95% CI	*P*‐value
Codominant model	PR	AA	23	1	—	Ref.
		AG	42	0.50	0.09–2.80	0.43
		GG	13	0.39	0.05–2.62	0.33
	SD	AA	10	1	—	Ref.
		AG	4	0.08	0.01–0.60	**0.01**
		GG	4	0.19	0.02–1.58	0.12
Recessive model	PR	AA	23	1	—	Ref.
		AG + GG	55	0.47	0.91–2.44	0.37
	SD	AA	10	1	—	Ref.
		AG + GG	8	0.12	0.20–0.72	**0.02**

Bold *P*‐values indicate *P* ≤ 0.05 from multivariate analysis by multinomial adjusted by age, TNM and PS‐ECOG. Reference category: DP, disease progression.

PR, partial response; SD, stable disease; Ref., reference category.

*According to RECIST criteria.

In addition, we evaluated the association between *EGF*+61 A>G genotypes and post‐TKI PFS and OS. No association was observed between *EGF*+61 A>G codominant model (AA vs. AG vs. GG; *P* = 0.562), recessive model (AA vs. AG + GG; *P* = 0.709) and dominant model (AA +AG vs. GG; *P* = 0.401) and post‐TKI PFS (Fig S1A–C, respectively). Likewise, no association was observed between *EGF*+61 A>G codominant model (AA vs. AG vs. GG; *P* = 0.493), recessive model (AA vs. AG

+GG; *P* = 0.318) and dominant model (AA + AG vs. GG; *P* = 0.342) and OS (Fig S1–F, respectively). Thus, the results show that the *EGF*+61 A>G polymorphism does not influence disease outcomes, both PFS and OS from NSCLC TKI‐treated patients harboring *EGFR*‐sensitizing mutations.

## Discussion

Molecular therapies have revolutionized clinical management in NSCLC patients, with particular emphasis on EGFR‐TKIs.^3^, ^4^, ^5^, ^15^, ^16^, ^17^ Herein, we report the impact of *EGF*+61 A>G genotype over ORR and DCR to first‐generation TKIs (erlotinib and gefitinib) on 111 *EGFR*‐mutated Brazilian lung adenocarcinoma patients.

The *EGF*+61 polymorphism (AG and AG + GG) was associated with stable disease in both univariate and multivariate in NSCLC patients treated with first‐generation TKIs harboring *EGFR*‐sensitizing mutations. Nevertheless, the SNP was not associated with ORR or disease outcome (progression‐free and overall survival). It is well known that *EGFR‐*sensitizing mutations can predict a response to first‐generation TKIs, and TK‐treated *EGFR‐*mutated patients can benefit from a substantial improvement in progression‐free and overall survival.^6^, ^17^ However, most patients will experience disease progression due to acquired resistance mechanisms.^7^ Currently, there is an effective third‐generation TKI, osimertinib, for patients harboring *EGFR*‐resistance mutations.^18^ Nonetheless, TKIs are considered high‐cost drugs for low‐middle income countries, and only first‐generation TKIs (erlotinib/gefitinib) are supported by the Brazilian public health system for *EGFR*‐mutated NSCLC patients. Thus, a biomarker for predicting both ORR and DCR in patients treated with first‐generation TKIs harboring *EGFR*‐sensitizing mutations would be valuable when financial resources are limited.

In conclusion, the present study revealed that the *EGF*+61 polymorphism (AG and AG + GG) was independently associated with stable disease, although the identification of this SNP cannot predict ORR, DCR or disease outcome in patients treated with first‐generation TKIs harboring *EGFR*‐sensitizing mutations.

## Disclosure

The authors declare that they have nothing to disclose.

## Supporting information


**Figure S1.** Survival analysis, according to *EGF*+61 genotypes. Progression‐free survival for codominant (**a**); recessive (**b**); and dominant (**c**) models. Overall survival for codominant (**c**); recessive (**d**); and dominant (**e**) models.

**Table S1.** Demographic and clinicopathological features of TKI‐treated NSCLC patients harboring *EGFR*‐sensitizing mutations (*n* = 111) and association with treatment response.
**Table S2.** Genotypic and allelic frequencies of TKI‐treated NSCLC patients harboring *EGFR* sensitizing mutations (*n* = 111).
