# Supplementary figures and images for: EGF+61 A>G polymorphism does not predict response to first‐generation EGFR tyrosine kinase inhibitors in lung cancer patients
Source: Thorac Cancer. 2020 Sep 3;11(10):2987–92. doi: 10.1111/1759-7714.13628 (PMC7529554; doi:10.1111/1759-7714.13628)

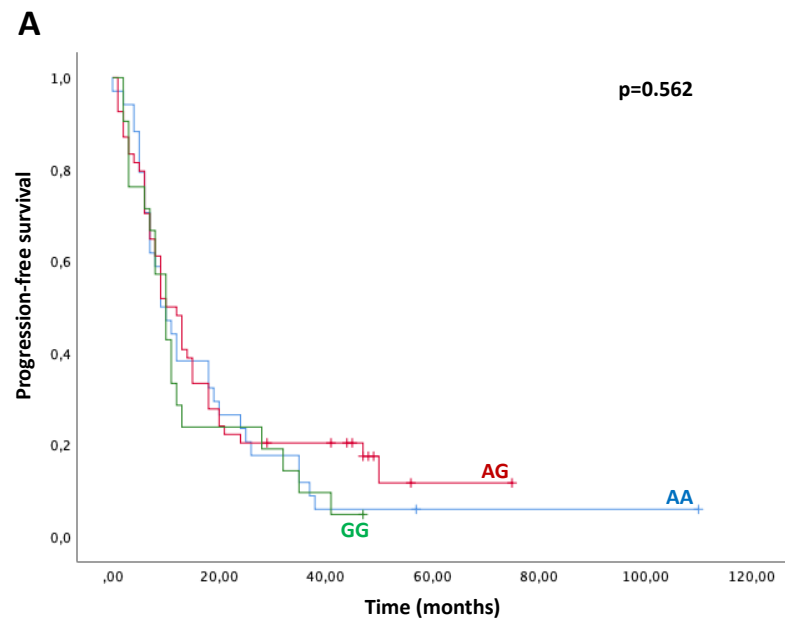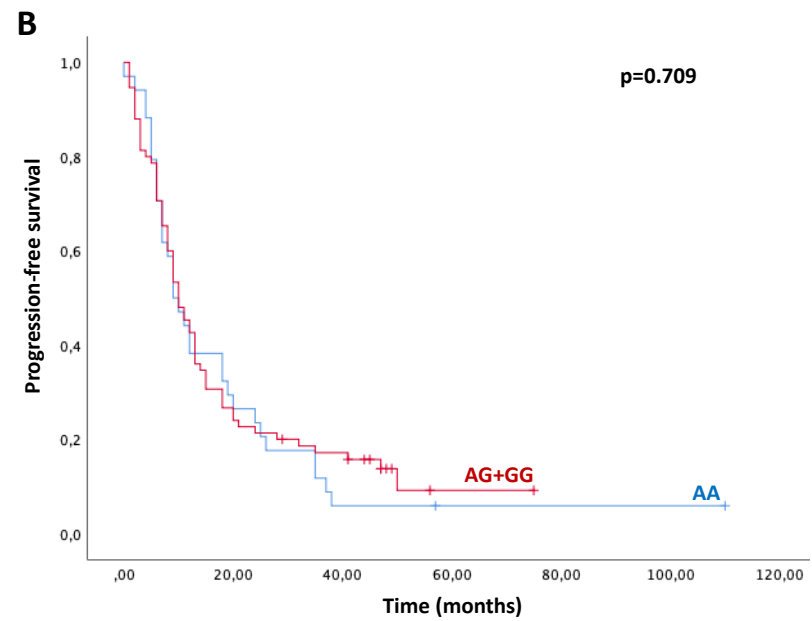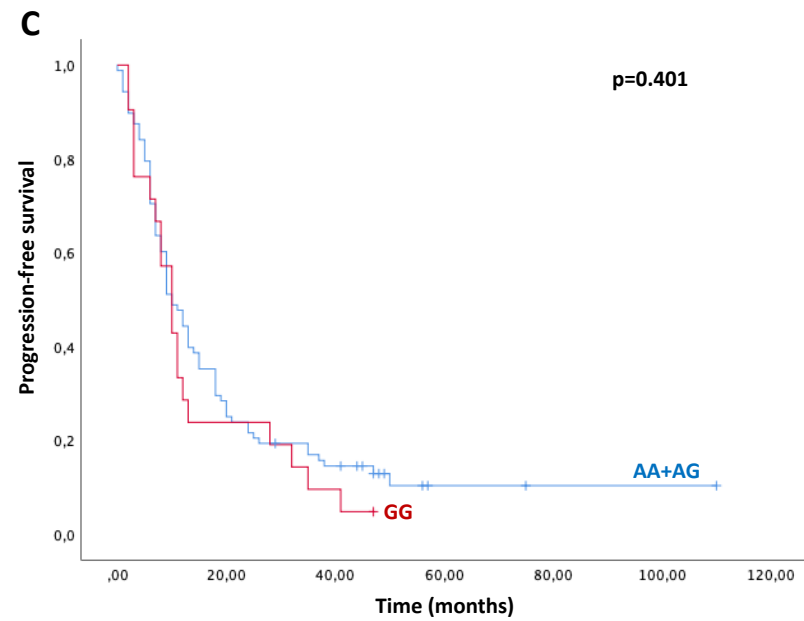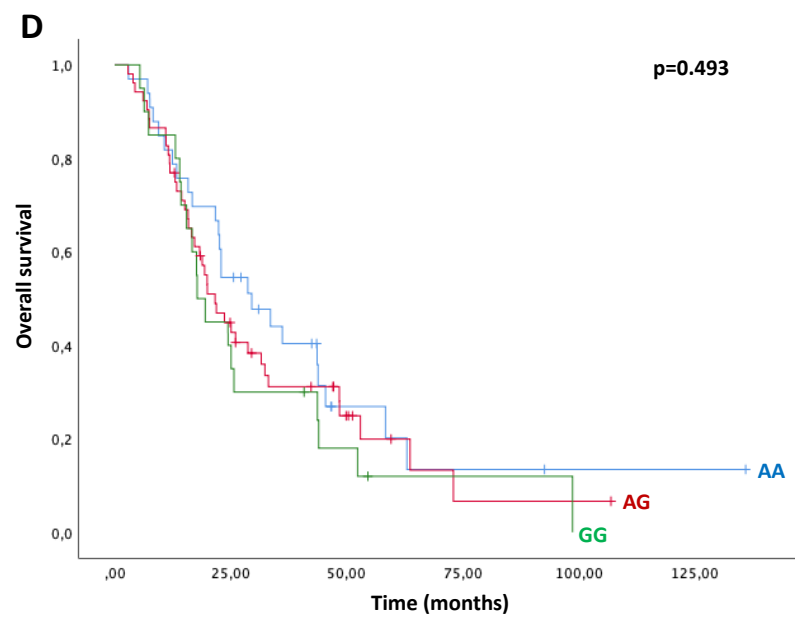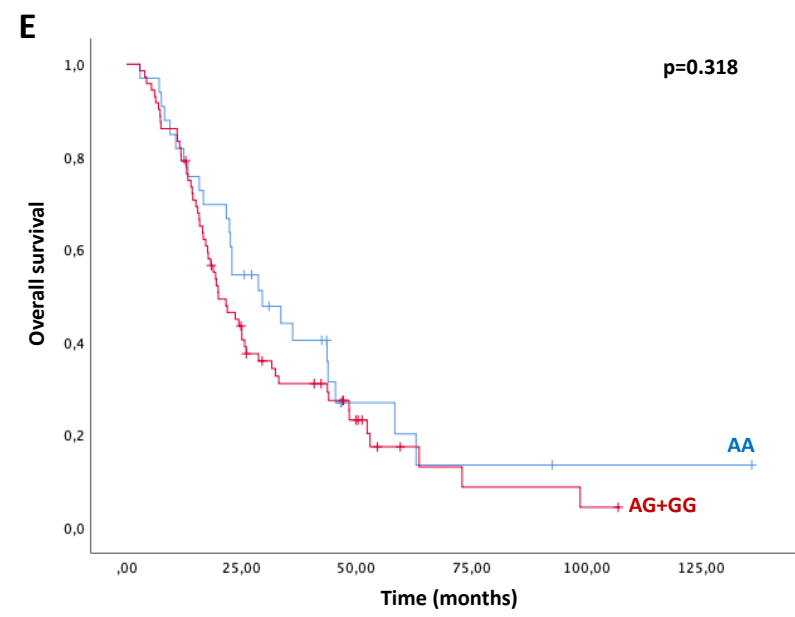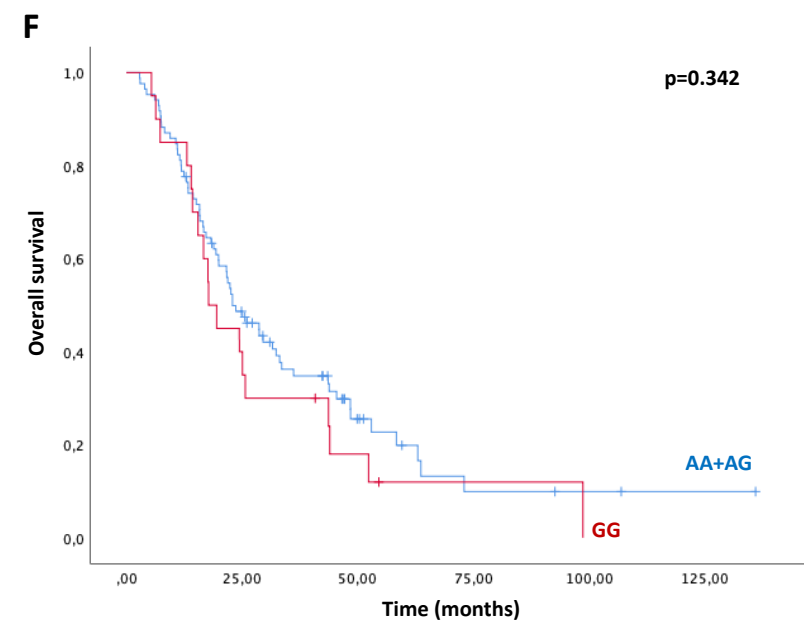

Supplement: Supplementary file 1 — Figure S1. Survival analysis, according to EGF+61 genotypes. Progression‐free survival for codominant (a); recessive (b); and dominant (c) models. Overall survival for codominant (c); recessive (d); and dominant (e) models. [file TCA-11-2987-s001.pdf]
