# Supplementary material for: EGF+61 A>G polymorphism does not predict response to first‐generation EGFR tyrosine kinase inhibitors in lung cancer patients
Source: Thorac Cancer. 2020 Sep 3;11(10):2987–92. doi: 10.1111/1759-7714.13628 (PMC7529554; doi:10.1111/1759-7714.13628)
Supplement: Supplementary file 2 — Table S1. Demographic and clinicopathological features of TKI‐treated NSCLC patients harboring EGFR‐sensitizing mutations (n = 111) and association with treatment response. Table S2. Genotypic and allelic frequencies of TKI‐treated NSCLC patients harboring EGFR sensitizing mutations (n = 111). [file TCA-11-2987-s002.pdf]

Supplementary Table 1: Demographic and clinical-pathological features of TKi-treated NSCLC patients harboring *EGFR*-sensitizing mutations (n=111) and association with treatment response.

**Characteristics**	**PR**		**SD**		**DP**		**p value**
Age							
≤63 years	45	57.7%	8	44.4%	5	33.3%	0.177
>63 years	33	42.3%	10	55.6%	10	66.7%	
Sex							
Male	32	41.0%	9	50.0%	6	40.0%	0.830
Female	46	59.0%	9	50.0%	9	60.0%	
Smoking status							
Never	45	58.4%	11	61.1%	9	60.0%	
Current	12	15.6%	2	11.1%	3	20.0%	0.969
Former	20	26.0%	5	27.8%	3	20.0%	
TNM stage							
I/II/III	6	7.7%	4	22.2%	2	13.3%	0.138
IV	72	92.3%	14	77.8%	13	86.7%	
ECOG PS							
0-1	51	69.9%	15	83.3%	12	80.0%	
2	18	24.7%	1	5.6%	1	6.7%	0.153
3-4	4	5.5%	2	11.1%	2	13.3%	
Weight loss in 6 months							
>10%	11	15.5%	4	22.2%	3	21.4%	
<10%	31	43.7%	9	50.0%	6	42.9%	0.863
None	29	40.8%	5	27.8%	5	35.7%	
Central nervous system metastasis							
Yes	23	29.5%	6	33.3%	3	20.0%	0.681
No	55	70.5%	12	66.7%	12	80.0%	

PR, partial response; SD, stable disease; DP, disease progression. P values indicate results from univariate analysis by χ2 test.

Supplementary Table 2: Genotypic and allelic frequencies of TKi-treated NSCLC patients harboring *EGFR*-sensitizing mutations (n=111).

**Characteristics**		**n = 111**	**Frequency***
Genotype (codominant model)	AA	35	31.5%
	AG	55	49.6%
	GG	21	18.9%
Genotype (recessive model)	AA	35	31.5%
	AG+GG	76	68.5%
Genotype (dominant model)	AA+AG	90	81.1%
	GG	21	
Allele frequency	A		56.3%
	G		43.7%

**excluding invalid values*
